# Comparision of biportal endoscopic and microscopic decompression in treatment of lumbar spinal stenosis: A comparative study protocol

**DOI:** 10.1097/MD.0000000000021309

**Published:** 2020-07-24

**Authors:** Jun Wu, Tao Guan, Feng Tian, Xueqi Liu

**Affiliations:** Departments of Orthopaedics, People's Hospital of Ningxia Hui Nationality Autonomous Region, Ningxia, China.

**Keywords:** lumbar spinal stenosis, microscopic bilateral decompression, percutaneous biportal endoscopic decompression, protocol

## Abstract

**Background:**

Microscopic bilateral decompression (MBD) has been suggested as an alternative to open laminectomy and fusion. Recently, percutaneous biportal endoscopic decompression (PBED) has begun to attract attention. The purpose of this retrospective study was to evaluate postoperative pain, functional disability, symptom reduction and satisfaction, and specific surgical parameters between the MBD and PBED techniques in patients with lumbar spinal stenosis (LSS).

**Methods:**

A retrospective review of LSS patients performed with MBD or PBED technique between May 2015 and June 2018 was conducted. Institutional review board approval in People's Hospital of Ningxia Hui Nationality Autonomous Region was obtained prior to conducting chart review and analysis. We received informed consent from all patients before surgery. The primary outcomes assessed were the preoperative to postoperative changes in leg/back pain and disability/function, patient satisfaction with the procedure, and postoperative quality of life. The secondary outcomes including duration of postoperative hospital stay, time to mobilization, postoperative analgesic use, complication rates, and baseline patient characteristics were prospectively collected.

**Results:**

The hypothesis was that the PBED technique would achieve better clinical outcomes as compared to the MBD technique in LSS.

## 1 Introduction

Lumbar spinal stenosis (LSS) is a prevalent and disabling condition in the aging population that often results in substantial physical burden for individuals with the disorder, and is associated with significant healthcare costs.^[1–3]^ An estimated 13% to 14% of those patients who seek help from a specialty physician, and 3% to 4% who seek care from a general practitioner for low back pain, are diagnosed with LSS.^[4,5]^ LSS is the most common degenerative disease indication for lumbar spinal surgery, with an estimated total annual inpatient expense of 1 billion dollars for over 30,000 surgical procedures performed.^[6–8]^

Traditionally, LSS is treated with an open decompressive laminectomy, a foraminotomy, or fusion. However, these methods may result in extensive bony destruction, and dissection and traction of the paraspinal muscles during surgery cause muscle atrophy, postoperative back pain, and post-spinal surgery syndrome.^[9–12]^ Recently, minimally invasive spinal surgical methods have developed to improve preservation of the surrounding normal anatomical structures, such as the muscles and ligaments. Microscopic bilateral decompression (MBD) has been suggested as an alternative to open laminectomy and fusion. Several studies have reported favorable longterm results, and the technique is currently considered the standard technique.^[13–18]^ However, vision is restricted and technical difficulties can arise in spite of using a microscope or uniportal spinal endoscope.^[19]^

Recently, percutaneous biportal endoscopic decompression (PBED) has been reported by several authors and has begun to attract attention. The method is based on the same operative technique as other surgical procedures, such as ipsilateral microscopic laminotomy and bilateral decompression, with patients in the prone position. Compared with open microscopic spinal surgery, the PBED technique can reduce muscle injury and allows excellent visualization of the contralateral traversing root.^[20–22]^ However, only a few studies have been published comparing the outcomes between MBD and PBED technique in patients with degenerative LSS.^[23–26]^ Due to a lack of direct comparison between the clinical outcomes of these 2 techniques in current literature, uncertainty remains regarding the superiority of either method. The purpose of this retrospective study was to evaluate postoperative pain, functional disability, symptom reduction and satisfaction, and specific surgical parameters between the 2 techniques. The hypothesis was that the PBED technique would achieve better clinical outcomes as compared to the MBD technique in LSS.

## 2 Materials and methods

### 2.1 Study design

A retrospective review of LSS patients performed with MBD or PBED technique between May 2015 and June 2018 was conducted with Institutional Review Board approval. All cases were performed by a single surgeon. Institutional review board approval in People's Hospital of Ningxia Hui Nationality Autonomous Region was obtained prior to conducting chart review and analysis (HZYY2004059). We received informed consent from all patients before surgery. This study was also registered in the Research Registry (researchregistry5702).

### 2.2 Participants

Inclusion criteria were the following: participants’ age between 30 and 80 years; degenerative LSS with radiating pain to lower extremities (score of visual analog scale >4); Definite lumbar central stenosis (Schizas grade ≥B) on magnetic resonance imaging; participants who were competent to understand the study protocol and able to be followed up regularly for 1 year after surgery; written informed consent. Exclusion criteria were the following: spondylolisthesis (≥Meyer grade II); history of lumbar spinal surgery for spinal stenosis or instability at the same level; stenosis caused by a herniated intervertebral disc; degenerative lumbar scoliosis (Cobb angle >20°); with other spinal diseases (eg, ankylosing spondylitis, spine tumor, fracture, or neurologic disorders); psychologic disorders; other disorders that the surgeon considered to make participation inappropriate.

### 2.3 Operative techniques

#### 2.3.1 MBD group

A 3 cm skin incision was made with a paramedian approach, slightly lateral to the midline. A muscle splitting technique using the microendoscopic tubular-retractor system left the midline structures, which support muscles and ligaments, intact. A tubular retractor was placed to create a surgical corridor and expose the lamina at the affected level. Muscle and other soft tissues covering the lamina and medial facet were resected. Unilateral laminectomy was performed with a high-speed burr, exposing the ligamentum flavum. Hypertrophied ligamentum flavum was excised with the Kerrison rongeur and curette.

#### 2.3.2 PBED group

Two separate 1 cm-sized skin incisions were made 1 cm above and below the disc space obliquely and 1 cm laterally from the midline. The first cranial portal was made as a viewing and continuous irrigation portal, and the second caudal portal was made in a more distal direction to be used as a working portal. A 0° arthroscope was inserted through the viewing portal, and a saline irrigation pump was connected and set to a pressure of 30 mmHg during the procedure. A continuous flow of saline irrigation by irrigation pump was essential to prevent excessive elevation of the epidural pressure. Using the working portal, conventional spinal surgical instruments and arthroscopic instruments were freely used in various angles. Ipsilateral decompression was executed by performing partial hemilaminectomy with a burr and the Kerrison rongeur until the superior edge of the deep part of the ligamentum flavum was exposed. The contralateral sublaminar space can be easily viewed by shifting the arthroscope and contralateral decompression was done by undercutting lamina with a burr and the Kerrison rongeur. The ligamentum flavum was carefully dissected from the dura and completely excised.

### 2.4 Clinical outcome measures

The primary outcomes assessed were the preoperative to postoperative changes in leg/back pain and disability/function, patient satisfaction with the procedure, and postoperative quality of life (Table 1). Pain was measured according to a self-assessment 10-point visual analog scale for leg pain only. Physical and mental health symptoms were measured using the Oswestry Disability Index and 12-Item Short Form Health Survey questionnaire. Patient satisfaction with the procedure was measured using a patient satisfaction index questionnaire. The secondary outcomes including duration of postoperative hospital stay, time to mobilization, postoperative analgesic use, complication rates, and baseline patient characteristics were prospectively collected. Duration of postoperative hospital stay was determined in hours from the time patients entered recovery until discharge. Time to mobilization was determined in hours from the time patients entered recovery until the medical notes documented they were able to “sit to stand” or “mobilize with supervision.”

**Table 1:**
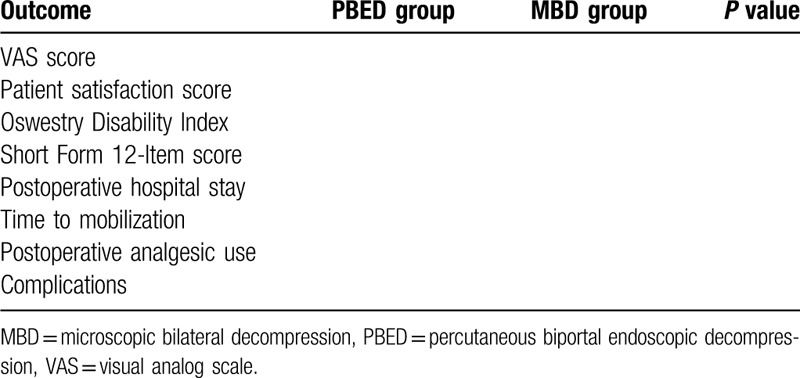
Postoperative outcomes.

### 2.5 Statistical analysis

All statistical analyses were performed using the SPSS ver. 18.0 (SPSS Inc., Chicago, IL). Values are presented as mean and standard deviation. Patient data were analyzed using the paired t-test. A *P* < .05 was regarded as statistically significant.

## 3 Discussion

The MBD technique allow adequate decompression preserving the contralateral facet joint, muscle, and posterior ligamentous complex while minimizing the ipsilateral facet joint disruption. Numerous studies concluded that facet preservation is the key factor to prevent postoperative spinal instability.^[27,28]^ Previous studies reported that MBD lessens operation time and blood loss than conventional open laminectomy, and also it yielded good long term outcome.^[19,20]^ However, there are some limitations in MBD. First, due to instrument entry through a small incision, access to the contralateral side is difficult. Second, the microscopy should be sometimes be excessively tilted in some cases. Third, the approach to the contralateral side is uneasy especially in the obese or heavy patients.^[29]^ Also, a minimal exposure may lead to inadequate decompression, resulting in remnant symptom or requiring reoperation.^[30,31]^ Finally, due to the steep learning curve, operation time could be prolonged. Recently multiple studies reported PBED, using 2 percutaneous portals ipsilateral to the lesion with continuous irrigation along, that allows similar decompression technique as MBD, using the state-of-art optical devices and light source.^[21,22]^ The purpose of this retrospective study was to evaluate postoperative pain, functional disability, symptom reduction and satisfaction, and specific surgical parameters between the 2 techniques. The limitations of our study included those inherent in any retrospective cohort study, including the possibility of selection or observational bias.

## Author contributions

Jun Wu and Tao Guan planned the study design and wrote the study protocol. Feng Tian and Xueqi Liu reviewed the study protocol. Jun Wu, Feng Tian and Xueqi Liu will recruit participants and collect data. Jun Wu wrote the manuscript. All of the authors have read, commented on, and contributed to the submitted manuscript.
